# Healthy Sleep Behaviors Reduce the Risk of Microvascular and Cardiovascular Complications in Patients With Type 2 Diabetes and Are Associated With Potential Serum Biomarkers: A UK Biobank Observational Cohort Study

**DOI:** 10.1111/1753-0407.70107

**Published:** 2025-06-29

**Authors:** Rui Lan, Lina Mao, Tingting Luo, Wenjin Luo, Yao Qin, Hanwen Ye, Jingbo Hu, Shuming Yang, Qifu Li, Zhihong Wang, Xiangjun Chen

**Affiliations:** ^1^ Department of Clinical Nutrition, Chongqing University Cancer Hospital, School of Medicine Chongqing University Chongqing China; ^2^ Department of Endocrinology The First Affiliated Hospital of Chongqing Medical University Chongqing China; ^3^ Department of Endocrinology The Affiliated Dazu's Hospital of Chongging Medical University, The People's Hospital of Dazu Chongqing China

**Keywords:** diabetes complications, diabetic cardiovascular complications, diabetic microvascular complications, mediating effects, sleep behavior, type 2 diabetes

## Abstract

**Background:**

The association of sleep behaviors with microvascular complications and cardiovascular outcomes in diabetic patients is not clear. Furthermore, serum biomarkers that can be used to evaluate this association have not been characterized. Therefore, this study investigated the association of the overall sleep score with the diabetic complications and the potential underlying serum metabolic biomarkers in patients with type 2 diabetes mellitus (T2DM).

**Methods:**

This prospective cohort study included 30 915 T2DM patients without complications from the UK Biobank. The sleep score of the participants was evaluated based on sleep behaviors such as sleep duration, insomnia, snoring, chronotype, and daytime sleepiness. The potential biomarkers, including cystatin C (Cys C), apolipoprotein A (Apo A), C‐reactive protein (CRP), albumin, and γ‐glutamyl transpeptidase (GGT), were also determined to evaluate their role as potential indicators of the association between the sleep score and the diabetic complications.

**Results:**

Participants with a healthy sleep score of 4–5 had lower risks of microvascular complications (HR = 0.80 [95% CI: 0.72, 0.89]) and cardiovascular outcomes (HR = 0.70 [95% CI: 0.61, 0.81]) compared to those with a sleep score of 0–1. Furthermore, cys C showed the best effects by explaining the associations of overall healthy sleep behaviors with microvascular complications and cardiovascular outcomes by 30.36% and 14.36%, respectively.

**Conclusions:**

Our data showed that healthy sleep behaviors were associated with a reduced risk of diabetic complications. Moreover, serum biomarkers of renal function, lipids, systemic inflammation, and hepatic function partially mediated the relationship between sleep behaviors and diabetic complications.

## Graphical abstract

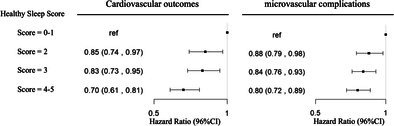

**Summary**

- Based on 30 915 T2D patients, this is the first large prospective study that demonstrated that healthy sleep behaviors were associated with a reduced risk of diabetic complications and cardiovascular outcomes.
- This is also the first study to use clinical biomarkers to determine the association between sleep behaviors and diabetic complications.
- Serum biomarkers such as Cys C, Apo A, CRP, albumin, and GGT were identified as potential biomarkers to determine the association of the sleep score with the diabetic microvascular and cardiovascular outcomes.
- Cys C showed the best effects by reducing the microvascular complications and cardiovascular outcomes by 30.36% and 14.36%, respectively.
- After adjusting for the confounding factors, participants with a healthy sleep score of 4–5 had lower risks of microvascular complications (HR = 0.80 [95% CI: 0.72, 0.89]) and cardiovascular outcomes (HR = 0.70 [95% CI: 0.61, 0.81]) compared to those with a poor sleep score of 0–1.

## 1 Introduction

Diabetes mellitus (DM) is a group of metabolic diseases characterized by persistent hyperglycemia. Estimates from the International Diabetes Federation Atlas (10th edition; 2021) showed that 1 in 10 individuals between 20 and 79 years of age were affected with DM globally [1, 2]. The relative risk of microvascular and cardiovascular complications is significantly higher in T2DM patients than in the non‐diabetic patients. About 50% of T2DM patients develop microvascular complications, and 27% develop cardiovascular complications [3]. Diabetic patients are associated with a high risk of adverse outcomes. The incidence rates of diabetes retinopathy(DR), diabetes neuropathy (DN), diabetic kidney disease (DKD), stroke, heart failure (HF), and coronary artery heart disease in diabetic patients are 34%, 31%–37%, 29%–61%, 8%–12%, 19%–26%, and 14%–21%, respectively [4].

In recent years, the importance of sleep in the management of patients with diabetes has garnered significant international attention and emphasis [5]. Previous studies have also shown that sleep behaviors such as sleep duration, insomnia, snoring, chronotype, and daytime sleepiness are associated with the risk of death in patients with diabetes [6, 7, 8, 9, 10, 11]. However, most of the studies have only assessed the relationship of individual sleep behaviors with diabetes, while a sleep behavior usually causes compensatory changes in other sleep behaviors. Therefore, it is important to evaluate the combination of various sleep behaviors [12], Previous studies have also suggested that joint assessment of multiple sleep behaviors may highlight their greater impact on human diseases [13].

In addition, there are a range of intermediate variables that are not known, such as Cys C, Apo A, albumin, CRP, and GGT. It is unclear whether and to what extent these potential biomarkers can mediate the association between sleep behavior and diabetes complications.

Therefore, in this study, we aimed to determine the potential relationship between healthy sleep behaviors and the risk of microvascular complications and cardiovascular outcomes in T2DM. First, we prospectively analyzed the joint relationship between individual sleep behaviors (sleep duration, insomnia, snoring, chronotype, and daytime sleepiness) and the incidences of total microvascular complications and cardiovascular outcomes in patients with T2DM from the UK Biobank. Furthermore, we analyzed a range of potential serum biomarkers to comprehensively evaluate the correlation between sleep behavior and diabetic complications.

## 2 Materials and Methods

### 2.1 Study Population

The UK Biobank is a biomedical database that contains data from a large prospective cohort study that was conducted with more than half a million participants from the UK between 2006 and 2010 [14]. The detailed design and methodology of the UK Biobank study have been described previously [15, 16]. The study was supported by the UK Biobank (ID 66536) and approved by the Northwest Multi‐Centre Research Ethics Committee (Ref: 13/NW/0382). All the participants provided written informed consent at the initial evaluation in the UK Biobank study.

**FIGURE 1:**
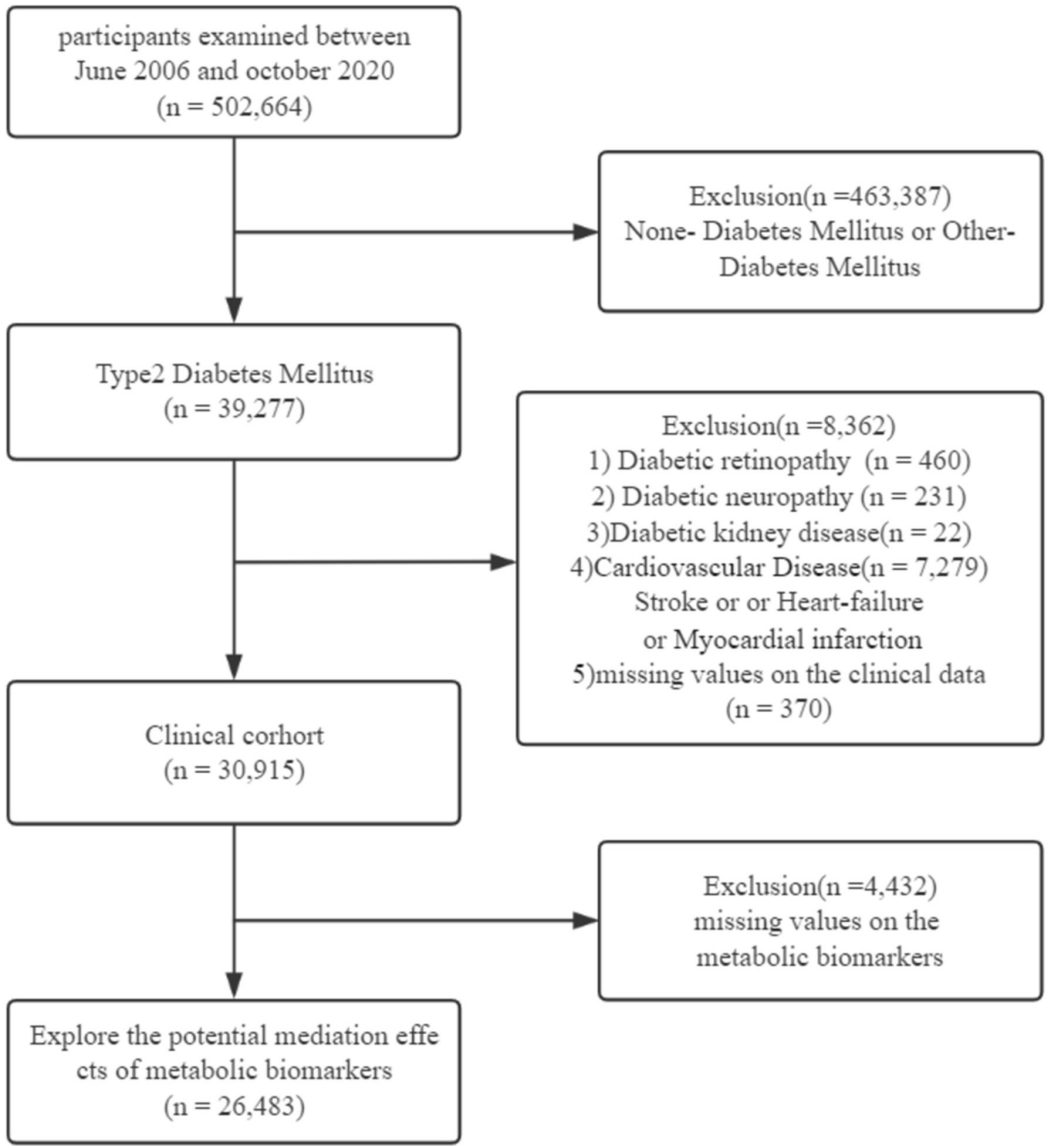
Flow diagram of selection of study population from the UK Biobank.

We excluded (1) participants without DM or those with DM types that were different from T2D (*n* = 463 387); (2) participants with DKD (*n* = 22), DN (*n* = 231), DR (*n* = 460), or cardiovascular disease (CVD) (*n* = 7279); and (3) participants without relevant missing clinical data (*n* = 370). Subsequently, we included 30 915 participants to analyze the relationship between the sleep behaviors and diabetic complications. For the study regarding potential mediating factors, we excluded participants without values for relevant metabolic biomarkers (*n* = 4432) and enrolled 26 483 participants for the final analysis (Figure 1).

### 2.2 Assessment of Sleep Behavior

Sleep behavior data was based on the self‐reported information collected at baseline from the study participants in the UK Biobank. Healthy sleep score was based on five sleep behaviors, which included sleep duration, insomnia, snoring, chronotype, and daytime sleepiness. The sleep score was estimated based on the methodology described in previous studies [12, 13, 17]. The sleep score was estimated as follows: Sleep duration was estimated by calculating the total daily sleep time over a 24‐h period and included the naps. Insomnia was estimated based on the responses of the participants to the following question: Do you experience difficulty in falling asleep at night or waking up in the middle of the night? The answer options were (1) never/rarely, (2) sometimes, and (3) usually. The presence of snoring was determined based on the answer of the participants to the following question: “Do your partner, relatives, or friends say that you snore?” The answer options were (1) yes or (2) no. Chronotype categorization of the patients was based on the answers to the following question: “Which of the following describes you best: (1) definitely a ‘morning’ person; (2) more ‘morning’ than ‘evening’; (3) more ‘evening’ than ‘morning’; or (4) definitely a ‘night’ person?.” Daytime sleepiness was evaluated by asking the participants to rate the frequency of dozing or falling asleep during the day and excluding intentional naps while working, reading, or driving. The answer options were as follows: (1) never/rarely, (2) sometimes, (3) frequently, and (4) all the time. If any of these factors vary significantly, participants are instructed to answer these questions based on their experiences over the past 4 weeks.

Healthy sleep behavior was defined as follows: (1) daily sleep duration of 7–8 h; (2) very low frequency or absence of insomnia symptoms (never, rarely or sometimes); (3) absence of snoring; (4) an early chronotype being “definitely morning” or “more morning than evening”; and (5) very low frequency or absence of daytime sleepiness (never, rarely, or sometimes). Each of the healthy sleep behaviors were scored as 1 point whereas others were scored as 0. The sleep score that was calculated by adding the score for each of the 5 sleep behaviors and ranged from 0 to 5. Higher sleep scores were indicative of an healthier sleep pattern. Based on the sleep scores, the study subjects were categorized into those with an “healthy sleep pattern” (sleep score of 4 or 5), “intermediate sleep pattern” (sleep score of 2 or 3), and “poor sleep pattern” (sleep score of 0 or 1).

### 2.3 Assessment of Circulating Biomarkers

Blood samples were collected from the study population and stored in the UK Biobank at −80°C and liquid nitrogen. The concentrations of several circulating biomarkers were estimated in samples that passed strict quality control, and the data was reported by the UK Biobank Biomarker Project [18]. To evaluate the mediating biomarkers in the relationship between sleep behavior and vascular complications, circulating biomarkers, including Cys C, Apo A, CRP, albumin, and GGT, were selected because they have not only been reported to be associated with sleep, but are also involved in biological processes that are important mechanisms for diabetic vascular complications, such as metabolism and inflammation‌ [19, 20, 21, 22, 23, 24].

### 2.4 Ascertainment of Outcomes

The cases of DR (ICD‐10: E113, E143, H280, H360), DN (ICD‐10: E114, E144, G590, G629, G632, G990), DKD (ICD‐10: E112, E142, N180, N181, N182, N183, N184, N185, N188, N189), and HF (ICD‐10: I69) were identified by linking the cohort database with the hospital inpatient admissions and death registries. The cases of stroke and myocardial infarction (MI) were identified using the UK Biobank database [25, 26]. Diabetic microvascular complications were defined as the occurrence of any one of DR, DKD, and/or DN. Diabetic cardiovascular outcomes were defined as the occurrence of any one of MI, stroke, or HF. The time of occurrence of these complications was calculated from the time of first occurrence.

### 2.5 Statistical Analysis

Statistical analysis was performed using the R software version 4.1.2 (http://www.R‐project.org; The R 121 Foundation). The follow‐up period was calculated from the day of recruitment until the occurrence of the diagnostic outcome or the last follow‐up, whichever occurred first. The categorical variables were encoded as values ranging from 0 to 1. The normally distributed continuous variables were represented as mean ± SD. The Cox proportional hazards model was used to determine the association between exposure to healthy sleep score and the diabetic complication outcomes, and the hazard ratios (HRs) and 95% confidence interval (CIs) were calculated.

We screened for confounding factors that were closely related to outcomes using previous literature and clinical findings [6, 13, 27, 28, 29]. The multivariate adjusted model was used to determine the association of potential confounders with the diabetic complications and the clinical outcomes. Age, sex, and ethnicity were adjusted in Model 1. Metabolic equivalent task (MET), Townsend Deprivation Index (TDI), alcohol consumption, and smoke status were further adjusted in Model 2. Model 3 was additionally adjusted for total triglyceride (TG), hypertension, and glycated hemoglobin (HbA1c). Furthermore, to ascertain the stability of the outcome, we further corrected for healthy diet, college, household income, and the use of lipid‐lowering, diabetes, and antihypertensive medications. We categorized diet into “healthy” and “unhealthy” based on a healthy diet score previously employed in several studies [30, 31]. This score reflects adherence to the AHA guidelines [32]. College means having a college or university degree. To avoid reverse causation, we excluded participants who developed microvascular complications or cardiovascular outcomes within 2 years of follow‐up [13, 27]. Moreover, the population‐attribution fractions (PAFs) were calculated to predict the proportion of microvascular complications and cardiovascular outcomes that could theoretically be avoided if all the participants showed healthy sleep behaviors. We also calculated weighted healthy sleep scores based on the five sleep behaviors using the following equation: weighted healthy sleep score = (*β*1 × factor 1 + *β*2 × factor 2 + … + *β*5 × factor 5) × (5/sum of the *β* coefficients). The *β* coefficients were derived from regression analyses of the associations between the five sleep behaviors and the primary outcomes [12, 13]. The weighted sleep score ranged from 0 to 5 points and considered the magnitude of the adjusted relative risk for each factor in each sleep pattern as a combination of five factors. The Kaplan–Meier estimates were used to calculate and illustrate cumulative survival rates. The statistical differences between groups were compared using the log‐rank test. Based on the previous reports regarding the potential causal pathways of microvascular and cardiovascular outcomes in T2D, several serum biomarkers from the UK Biobank were selected for the mediation analysis. *p* < 0.05 and *p* < 0.01 were considered statistically significant.

## 3 Results

### 3.1 Basic Characteristics of the Study Population

In the study population, we identified 4843 cases of microvascular complications and 2927 cases of cardiovascular outcomes. The median time to diabetic microvascular complications was 8.2 years, and for diabetic cardiovascular outcomes, it was 6.1 years. Table 1 shows the baseline characteristics of 30 915 T2D participants. The mean age of the 30 915 participants with T2D was 71.2 years, and 57.1% of the study participants were females. The study participants were categorized according to sleep scores into the following 4 categories: score 0–1, *n* = 2551 (8.3%); score 2, *n* = 8107 (26.2%); score 3, *n* = 11 603 (37.5%); and score 4–5, *n* = 8654 (28.0%). Participants with healthier sleep scores tended to be females, highly educated, low alcohol consumers and smokers, and those with healthier diet, higher physical activity, and lower body mass index (BMI). The baseline characteristics of the 26 484 participants are shown in Table S1.

**TABLE 1:** Baseline characteristics of 30 915 T2D participants according to the healthy sleep score.

Overall	Healthy sleep score					
	All	0–1	2	3	4–5	*p*
*N*	30 915	2551	8107	11 603	8654	
Females (%)	17 659 (57.1)	1399 (54.8)	4583 (56.5)	6604 (56.9)	5073 (58.6)	0.002
Age (year)	71.2 (7.5)	69.9 (7.6)	70.8 (7.5)	71.4 (7.5)	71.7 (7.5)	< 0.001
Ethnic White (%)	27 236 (88.1)	2221 (87.1)	7107 (87.7)	10 233 (88.2)	7675 (88.7)	0.071
Smoking (%)	3950 (12.8)	428 (16.8)	1228 (15.1)	1416 (12.2)	878 (10.1)	< 0.001
Alcohol (%)	21 293 (68.9)	1633 (64.0)	5545 (68.4)	8120 (70.0)	5995 (69.3)	< 0.001
Household income (mean [SD])	1.5 (2.0)	1.3 (1.9)	1.5 (2.0)	1.5 (2.0)	1.6 (2.1)	< 0.001
College (%)	7572 (24.5)	571 (22.4)	1859 (22.9)	2818 (24.3)	2324 (26.9)	< 0.001
TDI (mean [SD])	24 062 (77.8)	1850 (72.5)	6099 (75.2)	9098 (78.4)	7015 (81.1)	< 0.001
Healthy diet (%)	−0.6 (3.4)	0.1 (3.5)	−0.3 (3.5)	−0.7 (3.3)	−0.9 (3.3)	< 0.001
Activity_MET(%)						0.438
Low	7413 (24.0)	631 (24.7)	1977 (24.4)	2759 (23.8)	2046 (23.6)	
Medium	8892 (28.8)	754 (29.6)	2277 (28.1)	3332 (28.7)	2529 (29.2)	
High	14 610 (47.3)	1166 (45.7)	3853 (47.5)	5512 (47.5)	4079 (47.1)	
BMI (kg/m^2^)	31.1 (5.8)	33.2 (6.5)	31.9 (5.9)	31.0 (5.6)	29.9 (5.3)	< 0.001
Hypertension (%)	16 654 (53.9)	1499 (58.8)	4568 (56.3)	6231 (53.7)	4356 (50.3)	< 0.001
SBP (mmHg)	142.6 (17.8)	141.8 (17.9)	142.3 (17.5)	142.7 (17.8)	143.0 (17.9)	0.006
DBP (mmHg)	84.0 (9.9)	85.0 (9.9)	84.5 (10.0)	83.9 (9.9)	83.4 (9.9)	< 0.001
MABP (mmHg)	103.5 (11.2)	103.9 (11.3)	103.8 (11.2)	103.5 (11.2)	103.2 (11.2)	0.006
TC (mmol/L)	5.1 (1.2)	5.2 (1.2)	5.2 (1.3)	5.1 (1.2)	5.1 (1.2)	< 0.001
TG (mmol/L)	2.2 (1.3)	2.4 (1.3)	2.3 (1.3)	2.2 (1.3)	2.1 (1.2)	< 0.001
HDL‐C (mmol/L)	1.3 (0.3)	1.2 (0.3)	1.2 (0.3)	1.3 (0.3)	1.3 (0.3)	< 0.001
LDL‐C (mmol/L)	3.2 (0.9)	3.2 (0.9)	3.2 (0.9)	3.2 (0.9)	3.1 (0.9)	< 0.001
HbA1c (mmol/mol)	6.5 (1.2)	6.6 (1.3)	6.5 (1.2)	6.5 (1.2)	6.5 (1.2)	0.215
FBG (mmol/L)	6.7 (3.0)	6.8 (3.1)	6.7 (2.9)	6.7 (3.0)	6.7 (3.0)	0.517
Use of lipid‐lowing medication (%)	14 863 (48.1)	1224 (48.0)	3934 (48.5)	5652 (48.7)	4053 (46.8)	0.055
Use of antihypertensive medication (%)	14 034 (45.3)	1210 (47.4)	3836 (47.3)	5277 (45.5)	3711 (42.9)	< 0.001
Use of diabetes medication (%)	3071 (9.9)	223 (8.7)	747 (9.2)	1157 (10.0)	944 (10.9)	< 0.001

*Note:* Values are mean ± SD or *n* (%).

Abbreviations: BMI, body mass index; DBP, diastolic blood pressure; FBG, fasting blood glucose; HbA1c, glycated hemoglobin; HDL‐C, high‐density lipoprotein cholesterol; LDL‐C, low‐density lipoprotein cholesterol; MABP, mean arterial blood pressure, MET, metabolic equivalent task; SBP, systolic blood pressure; TC, total cholesterol; TDI, townsend deprivation index; TG, total triglyceride.

### 3.2 Association Between Sleep Score and Risk of Diabetic Complications

**FIGURE 2:**
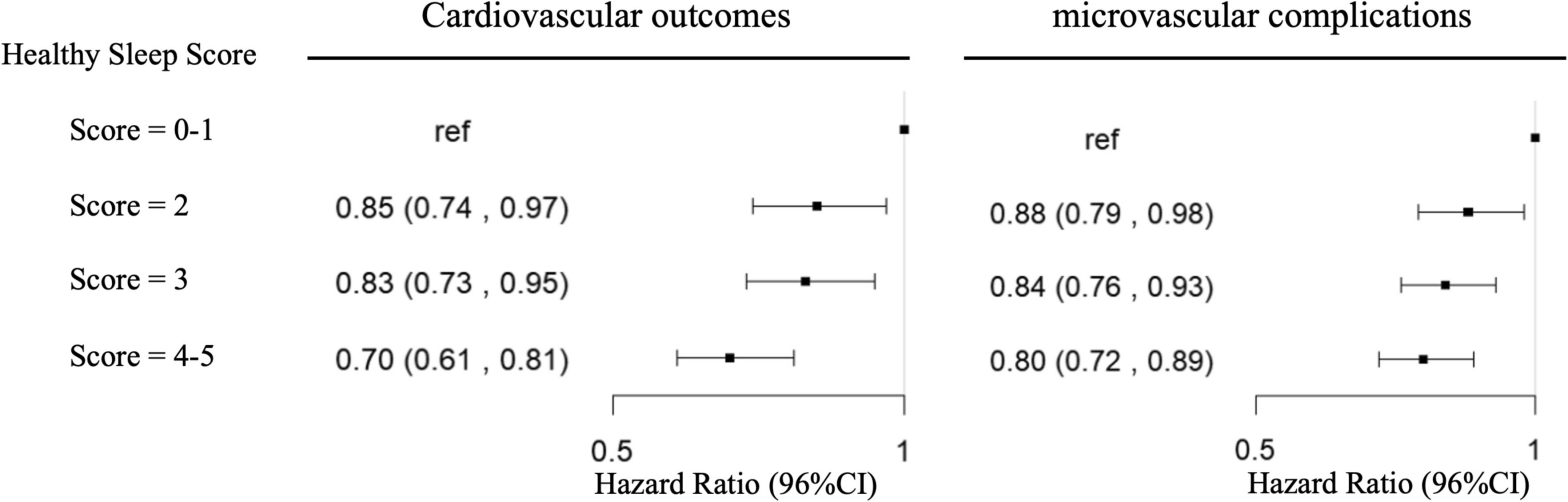
Multivariable‐adjusted incident risk of complications according to healthy sleep score among 30 915 T2D participants.

After multivariable adjustment, the risk of microvascular complications and cardiovascular outcomes was lower for participants with a healthy sleep score of 4–5 compared to those with a poor sleep score of 0–1. The HR (95% CI) values were 0.70 (0.61, 0.81) and 0.80 (0.72, 0.89), respectively (Table 2, Figure 2). When evaluated ordinally, each additional healthy sleep score was associated with a 9% lower risk of cardiovascular outcomes (HR for a one point higher healthy sleep score = 0.91 [95% CI: 0.87–0.94]), and a 6% lower risk of microvascular complications (HR = 0.94 [95% CI: 0.91–0.97]), (Table 2). Furthermore, the estimated PAFs indicated that having four or five healthy sleep behaviors was associated with a 5.3% (95% CI: 2.9%, 7.5%) decreased risk of diabetic microvascular complications and a 2.8% (95% CI: 0.9%, 4.6%) decreased risk of diabetic cardiovascular outcomes. This demonstrated that the probabilities of diabetic microvascular complications or cardiovascular outcomes were significantly low if all the participants were considered to be in the risk group with a healthy sleep score of 4–5. The Kaplan–Meier estimates for the survival cumulation also showed that higher stages of healthy sleep score had a higher survival rate of diabetes complications (Figure S1).

**TABLE 2:** HRs (95% CIs) of complications according to the healthy sleep score among 30 915 individuals with T2D participants.

	Healthy sleep score				*p* trend	HR continuous
	0–1	2	3	4–5		
*Microvascular complications*						
Model 1	Reference	0.85 (0.76, 0.95)	0.81 (0.73, 0.9)	0.75 (0.67, 0.84)	< 0.001	0.92 (0.89, 0.95)
Model 2	Reference	0.86 (0.77, 0.96)	0.83 (0.75, 0.92)	0.77 (0.70, 0.86)	< 0.001	0.93 (0.90, 0.96)
Model 3	Reference	0.88 (0.79, 0.98)	0.84 (0.76, 0.93)	0.80 (0.72, 0.89)	< 0.001	0.94 (0.91, 0.97)
PAF%				−5.3 (−7.50, −2.90)		
*Cardiovascular outcomes*						
Model 1	Reference	0.82 (0.72, 0.94)	0.79 (0.69, 0.90)	0.65 (0.57, 0.74)	< 0.001	0.88 (0.85, 0.92)
Model 2	Reference	0.84 (0.74, 0.96)	0.83 (0.73, 0.94)	0.69 (0.60, 0.79)	< 0.001	0.90 (0.86, 0.93)
Model 3	Reference	0.85 (0.74,0.97)	0.83 (0.73,0.95)	0.70 (0.61, 0.81)	< 0.001	0.91 (0.87, 0.94)
PAF%				−2.8 (−4.60, −0.90)		

*Note:* Model 1: Age (continuous), sex (male/female), ethnic (White/other). Model 2: Model 1 + Activity_MET (low/median/high), The Townsend Deprivation Index (continuous), alcohol consumption (special occasions only or never for yes/no), smoke status (yes/no). Model 3: Model 2 + TG (continuous), hypertension (yes/no), HbA1c (continuous). PAFs based on Model 3 were calculated to theoretically estimate the proportion of each outcome in this study population that could have been prevented if the population had ≥ 4–5 healthy sleep behaviors.

Abbreviations: CI, confidence interval; CVD, cardiovascular disease; HbA1c, glycated hemoglobin; HR, hazard ratio; MET, metabolic equivalent task; PAF, population‐attributable fraction; T2D, type 2 diabetes mellitus; TG, total triglyceride.

We then performed sensitivity analyses by further adjusting for confounding parameters such as healthy diet, college, household income, and the use of lipid‐lowering, diabetes, and antihypertensive medications, and observed a significant association between the sleep score and the incidence of diabetic complications (Table S2). As BMI is a key metabolic factor, previous studies have shown that BMI is associated not only with sleep disturbances but also with increased cysC; we further corrected for BMI to test the stability of the results [33]. The associations were not materially changed after further adjustment for BMI (microvascular complications, HR4–5 vs. 0–1: 0.84 [95% CI: 0.75, 0.95]; cardiovascular outcomes, HR4–5 vs. 0–1: 0.73 [95% CI: 0.63, 0.84]) (Table S3). The role of cysC in the association between sleep behavior and diabetes complications was diminished but still significantly associated. The percentages of risk reduction cysC accounted for were 17.75% (95% CI: 8.96, 64.03) for microvascular complications, while for cardiovascular outcomes, the percentages were 6.26% (95% CI: 3.13, 20.04) (Table S4). Furthermore, we also observed a significant association between the weighted sleep score and the incidence of diabetic complications (Table S5). Moreover, the sleep score showed a significant association with the incidence of diabetic complications even after excluding the incident cases that occurred within the first 2 years of follow‐up (Table S6). This provided evidence against reverse causation. We observed a significant relationship between sleep patterns and the risk of diabetes complications as shown in Table S7.

### 3.3 Association of Sleep Score With the Risk of Individual Diabetic Complications

Table S8 shows the association of sleep score with the risk of individual diabetic microvascular complications and cardiovascular outcomes. Compared to participants with a poor sleep score of 0–1, the HR (95% CI) values of participants with a healthy sleep score of 4–5 were 0.61 (0.48, 0.76), 0.72 (0.62, 0.84), 0.79 (0.63, 0.98), 0.64 (0.53, 0.76), and 0.73 (0.66, 0.82) for DN, DKD, stroke, HF, and CHD, respectively. The relationship between sleep score and DR did not show statistical significance in the multivariable‐adjusted model (HR [95% CI] = 1.06 [0.89, 1.26]).

Table S9 shows the correlation of individual sleep behaviors with the risk of diabetic cardiovascular outcomes and microvascular complications. In the multivariable correction model, no frequent insomnia, no frequent daytime sleepiness, and 7–8 h/day sleep were independently associated with a 13%, 26%, and 16% lower risk of cardiovascular outcomes, respectively, and a 6%, 15%, and 10% lower risk of microvascular complications, respectively.

### 3.4 Identification of Potential Serum Biomarkers to Analyze the Correlation Between Sleep Score and Diabetic Complications

Table 3 shows the potential biomarkers to analyze the correlation between sleep score and the incidence of diabetic complications. Cys C, Apo A, CRP, albumin, and GGT were identified as five potential biomarkers for evaluating the correlation between sleep score and the risk of diabetes complications. Based on the serum levels of Cys C, Apo A, CRP, albumin, and GGT, the risk of microvascular complications was reduced by 30.36%, 3.85%, 6.09%, 7.11%, and 3.05%, respectively, and the risk of cardiovascular outcomes was reduced by 14.36%, 5.29%, 10.85%, 6.28%, and 2.36%, respectively.

**TABLE 3:** Association of healthy sleep score with T2D microvascular complications and cardiovascular outcomes mediated by biomarkers among 26 483 individuals with T2D participants.

	Total effect			*p*	Natural direct effect			*p*	Natural indirect effect			*p*	Proportion eliminated	*p*
	*β*	Lower	Upper		*β*	Lower	Upper		*β*	Lower	Upper		% (95% CI)	
Microvascular complications														
Cys C (mg/L)	−0.0063	−0.0083	−0.0038	< 0.001	−0.0154	−0.0311	0.0005	0.08	−0.0216	−0.0367	−0.0052	< 0.001	30.36 (13.43, 108.55)	< 0.001
Apo A(g/L)	−0.0008	−0.0014	−0.0002	< 0.001	−0.0186	−0.0331	−0.0004	0.04	−0.0194	−0.034	−0.0010	0.04	3.85 (0.49, 27.98)	0.04
CRP (mg/L)	−0.0014	−0.0023	−0.0007	< 0.001	−0.0202	−0.0354	−0.0052	< 0.001	−0.0216	−0.0373	−0.0068	< 0.001	6.09 (2.74, 24.58)	< 0.001
Albumin (g/L)	−0.0015	−0.0024	−0.0006	< 0.001	−0.0194	−0.0381	−0.0032	0.04	−0.0209	−0.0397	−0.0041	0.02	7.11 (1.86, 24.98)	0.02
GGT (U/L)	−0.0007	−0.0014	−0.0001	< 0.001	−0.0192	−0.0377	−0.0053	0.04	−0.0199	−0.0383	−0.0062	0.04	3.05 (0.20, 15.22)	< 0.001
Cardiovascular outcomes														
Cys C (mg/L)	−0.0034	−0.0046	−0.0023	< 0.001	−0.0198	−0.0358	−0.0068	0.02	−0.0232	−0.0394	−0.0103	< 0.001	14.36 (8.41, 38.57)	< 0.001
Apo A (g/L)	−0.0012	−0.0018	−0.0006	< 0.001	−0.0211	−0.0353	−0.0074	< 0.001	−0.0223	−0.0370	−0.0086	< 0.001	5.29 (2.34, 14.26)	< 0.001
CRP (mg/L)	−0.0025	−0.0035	−0.0016	< 0.001	−0.0216	−0.0346	−0.0065	< 0.001	−0.0241	−0.0372	−0.0088	< 0.001	10.85 (6.24, 26.47)	< 0.001
Albumin (g/L)	−0.0015	−0.0024	−0.0005	< 0.001	−0.0220	−0.0363	−0.0092	< 0.001	−0.0234	−0.0377	−0.0106	< 0.001	6.28 (2.10, 15.76)	< 0.001
GGT (U/L)	−0.0006	−0.0012	−0.0001	< 0.001	−0.0229	−0.0404	−0.0099	< 0.001	−0.0235	−0.0414	−0.0105	< 0.001	2.36 (0.40, 6.67)	< 0.001

*Note:* Multivariable‐adjusted model: Age (continuous), sex (male/female), ethnic (White/other), Activity_MET (low/median/high), the Townsend Deprivation Index (continuous), alcohol consumption (special occasions only or never for yes/no), smoke status (yes/no), TG (continuous), hypertension (yes/no), HbA1c (continuous).

Abbreviations: Apo A, apolipoprotein A; CRP, C‐reactive protein; Cys C, cystatin C; GGT, γ‐glutamyl transpeptadase.

## 4 Discussion

This large prospective cohort study of patients with T2D showed that a healthy sleep score was significantly associated with a reduced risk of diabetic complications. Compared to individuals with a poor sleep score of 0–1, the risk of DN, DKD, stroke, HF, and CHD was reduced by 39%, 28%, 21%, 36%, and 27%, respectively, in those with a high sleep score of 4–5. Furthermore, we identified serum biomarkers such as Cys C, Apo A, CRP, albumin, and GGT as potential biomarkers that are associated with the sleep score and the diabetic microvascular complications and cardiovascular outcomes. Cys C was the most effective biomarker for evaluating the correlation of the sleep score with diabetic microvascular complications and cardiovascular outcomes.

Our data corroborates the findings from several previous reports regarding the relationship of individual sleep behavior parameters with the diabetic microvascular and cardiovascular complications. A previous study demonstrated that sleep duration and excessive daytime sleepiness were associated with an increased risk of DN [10]. Furthermore, short sleep duration was independently associated with DKD [34]. Insomnia is associated with a higher risk of cardiovascular outcomes, including stroke, HF, and CHD, especially when accompanied by short sleep duration [35]. Snoring was significantly associated with DKD and stroke risk [36, 37]. Poor sleep behaviors such as chronotype, insomnia, snoring, and daytime sleepiness were associated with the CVD events [38]. In this study, sleep score did not show significant correlation with DR. Previous studies have reported contradictory findings regarding the relationship of sleep behavior with the risk of developing DR. Simonson M et al., demonstrated that long sleep duration and insomnia were associated with an increased risk of DR [39]. However, other studies reported that sleep duration did not correlate significantly with the risk of DR [34, 39, 40, 41]. Moreover, another study performed multivariate analyses and reported that excessive daytime sleepiness was not associated with moderate DR [42]. Sleep and DR are complex physiological processes that involve interactions between several factors. Therefore, early clinical manifestations of DR are not obvious. This may be one of the reasons for not detecting the effects of low or moderate alterations in the sleep score on DR. Furthermore, the lack of statistical significance between sleep score and the risk of DR may also be due to a relatively small number of cases with DR outcomes. Moreover, differences in the study designs may contribute to contradictory findings reported in various studies. Therefore, well‐designed trials are necessary to confirm our findings in the future. It is worth noting that previous studies have suggested that sleep apnea is also a risk factor for diabetic complications [43, 44]. However, from a public health perspective, a simple scoring algorithm is necessary to easily interpret the epidemiological findings and translate into clinical practice because it is more informative for the general population.

Our mediation analysis deepens our comprehension regarding the decreased likelihood of diabetic microvascular and cardiovascular outcomes that is linked to healthy sleep behavior. Furthermore, our findings suggested that the association of healthy sleep score characterized by healthy sleep behaviors with the diabetic microvascular and cardiovascular outcomes can be mediated by improving renal function, lipid metabolism, systemic inflammation, and hepatic function. Cys C, a renal function‐related biomarker, showed the most significant effect in mediating the association of sleep score with both diabetic microvascular complications and cardiovascular outcomes.

Our study is the first large prospective cohort study that comprehensively examined the overall sleep behaviors in relation to diabetic microvascular and cardiovascular outcomes including DN, DKD, DR, stroke, HF, and CHD. We also demonstrated for the first time that the serum indicators such as Cys C, Apo A, CRP, albumin, and GGT were potential mediators of the association between sleep score and the diabetic complications. In our research, we highlight how the relationship between general sleep patterns and both diabetic microvascular and cardiovascular complications could potentially be accounted for by enhancements in glycemic control, liver functionality, lipid profiles, and overall systemic inflammation. The results of this study support potential interventions aimed at improving a wide range of sleep behaviors to prevent diabetic microvascular complications and cardiovascular outcomes risk.

Several mechanisms could explain the association between sleep behaviors and diabetic complications. Abnormal sleep behaviors were associated with circadian rhythm disorders, endocrine and metabolic dysregulation, hypoxemia, oxidative stress, and poor glycemic control, leading to microvascular and cardiovascular damage [36, 45, 46, 47, 48, 49, 50]. For example, sleep deprivation promotes the activation of the autonomic nervous system (characterized by increased secretion of catecholamines) and oxidative stress, both of which are associated with increased atherosclerosis, altering cardiovascular and microvascular structures [47]. Furthermore, sleep disturbances activation of pro‐inflammatory transcription factors such as nuclear factor‐κB (NF‐κB) exacerbates endothelial dysfunction and insulin resistance, thereby damaging the diabetic microvasculature [48]. Snoring causes intermittent hypoxemia and leads to increased sympathetic excitability and systemic oxidative stress and inflammation. This promotes overexpression of vascular endothelial factors and impairs the microvasculature [49, 50]. Daytime sleepiness also causes adverse cardiovascular effects through increased sympathetic activity [36].

To our knowledge, this is the first large prospective study of the association between overall sleep behaviors and diabetic complications. This is also the first study to use clinical biomarkers to determine the association between sleep behaviors and diabetic complications. Our study contributes significantly to advancing the knowledge regarding the impact of sleep score on the risk of microvascular complications and cardiovascular outcomes in diabetes. Our study also identified the importance of mediators. Prospective design, large sample size, and a long follow‐up period are other strengths of this study.

However, this study has several limitations. First, the data from self‐reported questionnaires to assess sleep behavior are vulnerable to recall bias. Furthermore, sleep behavior data was only collected at baseline and did not account for changes in sleep behavior over time. Second, the sleep score did not include all the sleep behaviors, including sleep apnea, which may additionally increase the risk of diabetic complications. Therefore, it is plausible that the risk was underestimated. Third, although we simultaneously evaluated the data regarding sleep behavior and biological mediators from the UK Biobank database, we did not estimate the causal relationship between sleep behavior and the biomarkers. Therefore, further research is required to verify this causal relationship in the future. Fourth, since the microvascular complications and stroke were identified only through hospital inpatient records and death registries, there may be underreporting of the cases. For example, the number of DR cases were small, potentially underestimating the association between sleep score and DR. Fifth, the biomarkers involved in this study cannot fully encapsulate all dimensions, such as cytokines and adipokines, which have been reported in previous research to be associated with sleep behavior or type 2 diabetes [51, 52]. Finally, this study used a multi‐factor corrected Cox regression method to obtain acceptable and credible results. However, the presence of other unmeasured or unknown residual confounding factors cannot be ruled out.

## 5 Conclusions

Our findings demonstrated that healthy sleep behaviors were associated with a reduced risk of diabetic complications and cardiovascular outcomes. Furthermore, we identified Cys C, Apo A, CRP, albumin, and GGT as potential biomarkers to determine the association of sleep behavior with the diabetic microvascular and cardiovascular complications. Our results provide a theoretical basis for the primary prevention of diabetes complications using interventions that improve sleep behavior.

## Author Contributions

**Rui Lan:** conceptualization, methodology, formal analysis, writing – original draft, writing – review and editing. **Lina Mao:** formal analysis, writing – review and editing. **Tingting Luo:** writing – original draft, writing – review and editing. **Wenjin Luo:** investigation, data curation. **Yao Qin:** formal analysis. **Hanwen Ye:** formal analysis. **Jingbo Hu:** investigation, data curation, writing – review and editing. **Shuming Yang:** funding acquisition. **Qifu Li:** funding acquisition. **Zhihong Wang:** conceptualization, methodology, investigation, data curation, writing – review and editing, funding acquisition, supervision, project administration. **Xiangjun Chen:** investigation, data curation, writing – review and editing, funding acquisition, manuscript revise.

## Conflicts of Interest

The authors declare no conflicts of interest.

## Supporting information


**Figure S1.** Kaplan–Meier curves for complications in 30 915 T2D participants. (a) Microvascular complications; (b) cardiovascular outcomes.

**Table S1.** Baseline characteristics of 26 483 T2DM participants according to the healthy sleep score.
**Table S2.** Associations between the healthy sleep score and incident microvascular complications or cardiovascular outcomes after further adjusting for confounders.
**Table S3.** HRs (95% CIs) of complications according to the healthy sleep score among 30 915 individuals with T2D participants.
**Table S4.** Association of healthy sleep score with T2D microvascular complications and cardiovascular outcomes mediated by biomarkers among 26 483 individuals with T2D participants.
**Table S5.** Multivariable‐adjusted HRs (95% CIs) for incident microvascular complications or cardiovascular outcomes among 30 915 UK Biobank T2DM participants by different weighted healthy sleep score.
**Table S6.** Associations between healthy sleep score and incident microvascular complications or cardiovascular outcomes after excluding incident cases occurred in the first 2 years of follow‐up.
**Table S7.** Multivariable‐adjusted HRs (95% CIs) for incident microvascular complications or cardiovascular outcomes among 30 915 UK Biobank T2DM participants by different Sleep pattern.
**Table S8.** Associations between healthy sleep score and incident individual microvascular complication or cardiovascular outcome among 30 915 UK Biobank T2DM participants.
**Table S9.** HRs of complications by individual healthy sleep behaviors among 30 915 UK Biobank T2DM participants.

## Data Availability

The individual participant data collected for the current study can not be shared without UK Biobank's explicit written approval.
