# Supplementary figures and images for: Healthy Sleep Behaviors Reduce the Risk of Microvascular and Cardiovascular Complications in Patients With Type 2 Diabetes and Are Associated With Potential Serum Biomarkers: A UK Biobank Observational Cohort Study
Source: J Diabetes. 2025 Jun 29;17(7):e70107. doi: 10.1111/1753-0407.70107 (PMC12206587; doi:10.1111/1753-0407.70107)

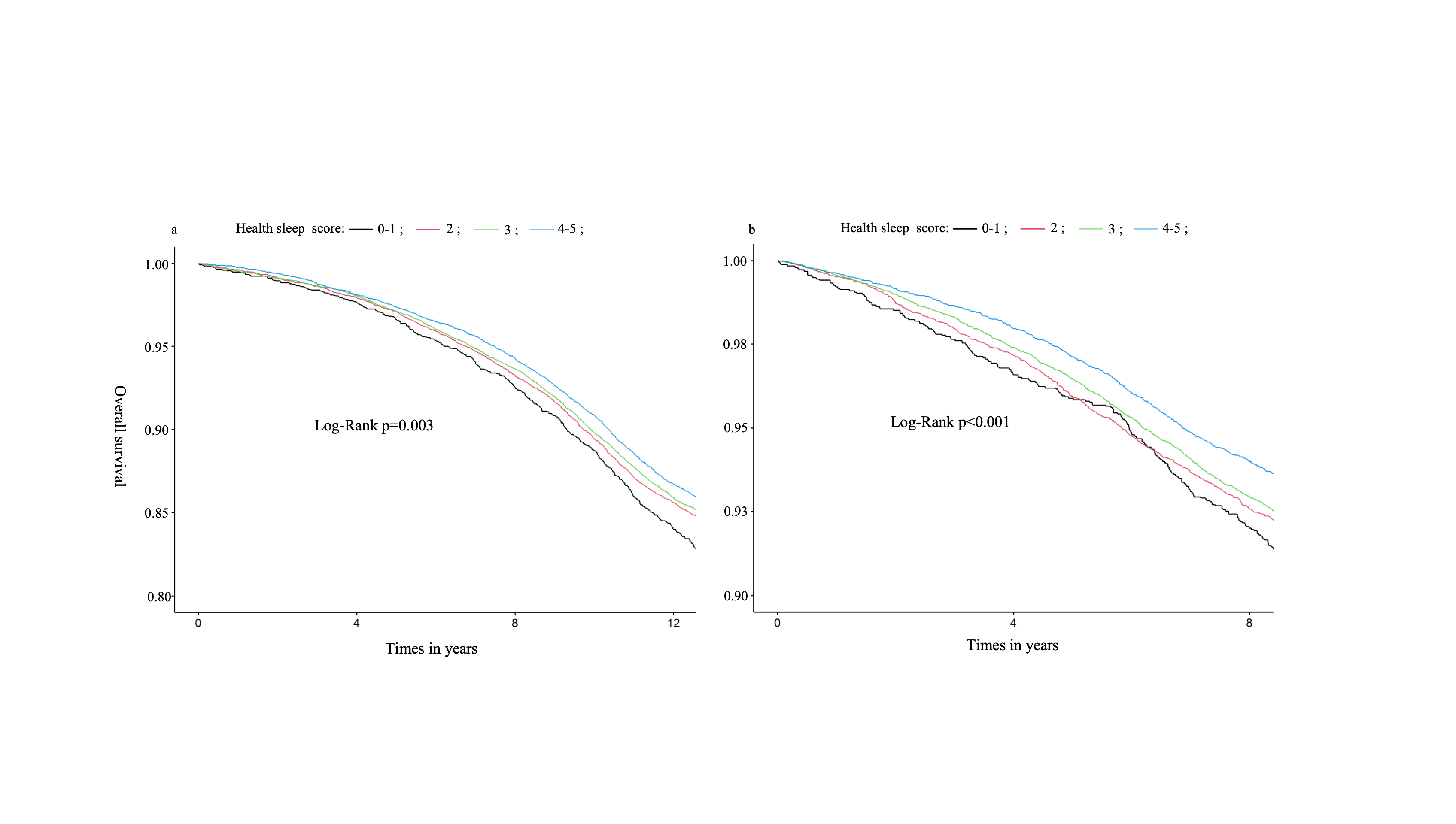

Supplement: Supplementary file 1 — Figure S1. Kaplan–Meier curves for complications in 30 915 T2D participants. (a) Microvascular complications; (b) cardiovascular outcomes. [file JDB-17-e70107-s002.png]
