# Supplementary material for: Healthy Sleep Behaviors Reduce the Risk of Microvascular and Cardiovascular Complications in Patients With Type 2 Diabetes and Are Associated With Potential Serum Biomarkers: A UK Biobank Observational Cohort Study
Source: J Diabetes. 2025 Jun 29;17(7):e70107. doi: 10.1111/1753-0407.70107 (PMC12206587; doi:10.1111/1753-0407.70107)
Supplement: Supplementary file 2 — Table S1. Baseline characteristics of 26 483 T2DM participants according to the healthy sleep score. Table S2. Associations between the healthy sleep score and incident microvascular complications or cardiovascular outcomes after further adjusting for confounders. Table S3. HRs (95% CIs) of complications according to the healthy sleep score among 30 915 individuals with T2D participants. Table S4. Association of healthy sleep score with T2D microvascular complications and cardiovascular outcomes mediated by biomarkers among 26 483 individuals with T2D participants. Table S5. Multivariable‐adjusted HRs (95% CIs) for incident microvascular complications or cardiovascular outcomes among 30 915 UK Biobank T2DM participants by different weighted healthy sleep score. Table S6. Associations between healthy sleep score and incident microvascular complications or cardiovascular outcomes after excluding incident cases occurred in the first 2 years of follow‐up. Table S7. Multivariable‐adjusted HRs (95% CIs) for incident microvascular complications or cardiovascular outcomes among 30 915 UK Biobank T2DM participants by different Sleep pattern. Table S8. Associations between healthy sleep score and incident individual microvascular complication or cardiovascular outcome among 30 915 UK Biobank T2DM participants. Table S9. HRs of complications by individual healthy sleep behaviors among 30 915 UK Biobank T2DM participants. [file JDB-17-e70107-s001.docx]

Supplementary Table1. Baseline Characteristics of 26,483 T2DM participants according to the healthy sleep score.

|  | **Healthy Sleep Score** | | | | |  |
| --- | --- | --- | --- | --- | --- | --- |
| Overall | all | 0-1 | 2 | 3 | 4-5 | p |
| N | 26484 | 2179 | 6936 | 9983 | 7386 |  |
| Females (%) | 15168 ( 57.3 ) | 1206 ( 55.3 ) | 3922 ( 56.5 ) | 5690 ( 57.0 ) | 4350 ( 58.9 ) | 0.005 |
| Age (year) | 71.3 ( 7.5 ) | 70.0 ( 7.6 ) | 70.8 ( 7.5 ) | 71.5 ( 7.5 ) | 71.8 ( 7.5 ) | <0.001 |
| Ethnic.White (%) | 23389 ( 88.3 ) | 1894 ( 86.9 ) | 6090 ( 87.8 ) | 8834 ( 88.5 ) | 6571 ( 89.0 ) | 0.027 |
| Smoking (%) | 3394 ( 12.8 ) | 359 ( 16.5 ) | 1056 ( 15.2 ) | 1229 ( 12.3 ) | 750 ( 10.2 ) | <0.001 |
| Alcohol (%) | 16893 ( 63.8 ) | 1404 ( 64.4 ) | 4413 ( 63.6 ) | 6335 ( 63.5 ) | 4741 ( 64.2 ) | 0.692 |
| Household.income( mean ( SD ) ) | 1.5 ( 2.0 ) | 1.3 ( 1.9 ) | 1.5 ( 2.0 ) | 1.5 ( 2.0 ) | 1.6 ( 2.1 ) | <0.001 |
| College (%) | 6499 ( 24.5) | 498 ( 22.9) | 1591 ( 22.9) | 2420 ( 24.2) | 1990 ( 26.9) | <0.001 |
| TDI ( mean ( SD ) ) | -0.6 ( 3.4 ) | 0.1 ( 3.5 ) | -0.4 ( 3.4 ) | -0.7 ( 3.3 ) | -0.9 ( 3.2 ) | <0.001 |
| Healthy diet ( % ) | 20618 ( 77.9 ) | 1581 ( 72.6 ) | 5229 ( 75.4 ) | 7827 ( 78.4 ) | 5981 ( 81.0 ) | <0.001 |
| Activity_MET(%) |  |  |  |  |  | 0.512 |
| Low | 6351 ( 24.0 ) | 543 ( 24.9 ) | 1699 ( 24.5 ) | 2372 ( 23.8 ) | 1737 ( 23.5 ) |  |
| Medium | 7626 ( 28.8 ) | 638 ( 29.3 ) | 1949 ( 28.1 ) | 2882 ( 28.9 ) | 2157 ( 29.2 ) |  |
| High | 12507 ( 47.2 ) | 998 ( 45.8 ) | 3288 ( 47.4 ) | 4729 ( 47.4 ) | 3492 ( 47.3 ) |  |
| BMI (kg/m2) | 31.2 ( 5.8 ) | 33.3 ( 6.5 ) | 31.9 ( 5.9 ) | 31.0 ( 5.6 ) | 30.0 ( 5.3 ) | <0.001 |
| Hypertension ( % ) | 14325 ( 54.1 ) | 1287 ( 59.1 ) | 3922 ( 56.5 ) | 5366 ( 53.8 ) | 3750 ( 50.8 ) | <0.001 |
| SBP (mmHg) | 142.6 ( 17.7 ) | 141.6 ( 17.9 ) | 142.3 ( 17.5 ) | 142.6 ( 17.7 ) | 143.1 ( 18.0 ) | 0.004 |
| DBP (mmHg) | 84.0 ( 9.9 ) | 84.9 ( 9.9 ) | 84.5 ( 9.9 ) | 84.0 ( 9.9 ) | 83.4 ( 9.9 ) | <0.001 |
| MABP (mmHg) | 103.6 ( 11.2 ) | 103.8 ( 11.3 ) | 103.8 ( 11.2 ) | 103.5 ( 11.2 ) | 103.3 ( 11.2 ) | 0.032 |
| FBG (mmol/L) | 6.7 ( 3.0 ) | 6.8 ( 3.1 ) | 6.7 ( 2.9 ) | 6.7 ( 3.0 ) | 6.8 ( 3.0 ) | 0.527 |
| TC (mmol/L) | 5.1 ( 1.2 ) | 5.2 ( 1.2 ) | 5.1 ( 1.3 ) | 5.1 ( 1.2 ) | 5.0 ( 1.2 ) | <0.001 |
| TG (mmol/L) | 2.2 ( 1.3 ) | 2.3 ( 1.3 ) | 2.3 ( 1.3 ) | 2.2 ( 1.2 ) | 2.1 ( 1.2 ) | <0.001 |
| HDL (mmol/L) | 1.2 ( 0.3 ) | 1.2 ( 0.3 ) | 1.2 ( 0.3 ) | 1.2 ( 0.3 ) | 1.3 ( 0.3 ) | <0.001 |
| LDL (mmol/L) | 3.2 ( 0.9 ) | 3.2 ( 0.9 ) | 3.2 ( 1.0 ) | 3.2 ( 0.9 ) | 3.1 ( 0.9 ) | <0.001 |
| HbA1c (mmol/mol) | 6.5 ( 1.2 ) | 6.5 ( 1.3 ) | 6.5 ( 1.2 ) | 6.5 ( 1.2 ) | 6.5 ( 1.2 ) | 0.424 |
| Apolipoprotein A(g/L) | 1.4 ( 0.2 ) | 1.4 ( 0.2 ) | 1.4 ( 0.2 ) | 1.4 ( 0.2 ) | 1.4 ( 0.3 ) | <0.001 |
| CRP (mg/L) | 3.7 ( 5.1 ) | 4.8 ( 5.6 ) | 4.1 ( 5.4 ) | 3.7 ( 5.1 ) | 3.2 ( 4.5 ) | <0.001 |
| Albumin (g/L) | 45.1 ( 2.7 ) | 44.8 ( 2.8 ) | 45.0 ( 2.8 ) | 45.1 ( 2.7 ) | 45.2 ( 2.7 ) | <0.001 |
| Cystatin C (mg/L) | 0.9 ( 0.2 ) | 1.0 ( 0.2 ) | 0.9 ( 0.2 ) | 0.9 ( 0.1 ) | 0.9 ( 0.1 ) | <0.001 |
| Total bilirubin(μmol/L) | 8.9 ( 4.3 ) | 8.4 ( 3.9 ) | 8.8 ( 4.3 ) | 8.9 ( 4.4 ) | 9.1 ( 4.5 ) | <0.001 |
| GGT (U/L) | 52.2 ( 56.8 ) | 59.6 ( 68.6 ) | 55.5 ( 61.6 ) | 51.6 ( 53.4 ) | 47.7 ( 52.1 ) | <0.001 |
| Use of lipid-lowing medication ( % ) | 12885 ( 48.7 ) | 1054 ( 48.4 ) | 3386 ( 48.8 ) | 4921 ( 49.3 ) | 3524 ( 47.7 ) | 0.221 |
| Use of antihypertensive medication( % ) | 12160 ( 45.9 ) | 1051 ( 48.2 ) | 3327 ( 48.0 ) | 4556 ( 45.6 ) | 3226 ( 43.7 ) | <0.001 |
| Use of diabetes medication ( % ) | 2586 ( 9.8 ) | 192 ( 8.8 ) | 629 ( 9.1 ) | 968 ( 9.7 ) | 797 ( 10.8 ) | 0.002 |

Values are mean±SD or n (%)

TDI: townsend deprivation index. MET:metabolic equivalent task. SBP: systolic blood pressure, DBP: diastolic blood pressure, BMI: body mass index,FPG: fasting plasma glucose, HbA1c: glycated hemoglobin, TG: total triglyceride, TC: total cholesterol, HDL-C: high densitylipoprotein cholesterol, LDL-C: low density lipoprotein cholesterol, CRP, C-reactive protein, GGT,γ-glutamyl transpeptadase.

Supplementary Table2. Associations between the healthy sleep score and incident microvascular complications or cardiovascular outcomes after further adjusting for confounders.

|  | **Healthy Sleep Score** | | | |  |  |
| --- | --- | --- | --- | --- | --- | --- |
|  | 0–1 | 2 | 3 | 4–5 | P trend | HR continuous |
| **Microvascular complications** |  |  |  |  |  |  |
| Model3 | Ref | 0.88 (0.79 , 0.98) | 0.84 (0.76 , 0.93) | 0.80 (0.72 , 0.89) | <0.001 | 0.94 (0.91 , 0.97) |
| Model3+Healthy diet+ Household.income+College | Ref | 0.88(0.79 , 0.98) | 0.85(0.76 , 0.94) | 0.80(0.72 , 0.90) | <0.001 | 0.94 (0.91 , 0.97) |
| Model3+Healthy diet+College+ Household.income+Use of lipid-lowing, diabetes, antihypertensive,medication | Ref | 0.87(0.78 , 0.97) | 0.84(0.75 , 0.93) | 0.79(0.71 , 0.88) | <0.001 | 0.94 (0.91 , 0.97) |
| **Cardiovascular outcomes** |  |  |  |  |  |  |
| Model3 | Ref | 0.85 (0.74 , 0.97) | 0.83 (0.73 , 0.95) | 0.70 (0.61 , 0.81) | <0.001 | 0.91 (0.87 , 0.94) |
| Model3+Healthy diet+ Household.income+College | Ref | 0.86(0.75 , 0.98) | 0.84(0.74 , 0.96) | 0.71(0.62 , 0.82) | <0.001 | 0.91 (0.87 , 0.95) |
| Model3+Healthy diet+College+ Household.income+Use of lipid-lowing,diabetes, antihypertensive,medication | Ref | 0.86(0.75 ,0.98) | 0.84(0.74 , 0.96) | 0.71(0.62 ,0.82) | <0.001 | 0.91 (0.87 , 0.94) |

Model3: Age (continuous), Sex (male/female), Ethnic (Whiter/other), Activity_MET (low/median/high), The Townsend Deprivation Index (continuous), Alcohol consumption (Special occasions only or Never for yes/no), Smoke status (yes/no),TG(continuous), Hypertension (yes/no), Hba1c (continuous).

Supplementary Table3: HRs (95% CIs) of complications according to the healthy sleep Score among 30,915 individuals with T2D participants.

|  | **Healthy Sleep Score** | | | |  |  |
| --- | --- | --- | --- | --- | --- | --- |
|  | 0–1 | 2 | 3 | 4–5 | P trend | HR continuous |
| **Microvascular complications** |  |  |  |  |  |  |
| Model3+BMI | Ref | 0.90(0.81,1.00) | 0.87(0.79,0.97) | 0.84(0.75,0.93) | 0.002 | 0.95(0.92,0.98) |
| **Cardiovascular outcomes** |  |  |  |  |  |  |
| Model3+BMI | Ref | 0.88(0.77,1.01) | 0.89(0.78,1.01) | 0.76(0.66,0.88) | <0.001 | 0.93(0.89,0.96) |

Model3: Age (continuous), Sex (male/female), Ethnic (Whiter/other), Activity_MET (low/median/high), The Townsend Deprivation Index (continuous), Alcohol consumption (Special occasions only or Never for yes/no), Smoke status (yes/no),TG(continuous), Hypertension (yes/no), HbA1c (continuous).

Supplementary Table4. Association of healthy sleep Score with T2D microvascular complications and cardiovascular outcomes mediated by biomarkers among 26,483 individuals with T2D participants.

|  | **Total effect** | | |  | **Natural direct effect** | | |  | **Natural indirect effect** | | |  | **Proportion eliminated** |  |
| --- | --- | --- | --- | --- | --- | --- | --- | --- | --- | --- | --- | --- | --- | --- |
|  | Beta | Lower | Upper | p | Beta | Lower | Upper | p | Beta | Lower | Upper | p | % (95%CI) | p |
| **Microvascular  complications** |  |  |  |  |  |  |  |  |  |  |  |  |  |  |
|  |  |  |  |  |  |  |  |  |  |  |  |  |  |  |
| Cys C (mg/L) | -0.0013 | -0.0020 | -0.0007 | <0.001 | -0.0059 | -0.0102 | -0.0007 | 0.020 | -0.0072 | -0.0113 | -0.002 | <0.001 | 17.75(8.96 ,64.03) | <0.001 |
| **Cardiovascular  outcomes** |  |  |  |  |  |  |  |  |  |  |  |  |  |  |
|  |  |  |  |  |  |  |  |  |  |  |  |  |  |  |
| Cys C (mg/L) | -0.0005 | -0.0008 | -0.0003 | <0.001 | -0.0067 | -0.0109 | -0.0027 | <0.001 | -0.0072 | -0.0115 | -0.0033 | <0.001 | 6.26(3.13 ,20.04) | <0.001 |

Multivariable-adjusted Model:Age (continuous), Sex (male/female), Ethnic (Whiter/other), Activity_MET (low/median/high), The Townsend Deprivation Index (continuous), Alcohol consumption (Special occasions only or Never for yes/no), Smoke status (yes/no),TG(continuous), Hypertension (yes/no), HbA1c (continuous), BMI(continuous).

Supplementary Table5. Multivariable-adjusted HRs (95%CIs) for incident microvascular complications or cardiovascular outcomes among 30,915 UK Biobank T2DM participants by different weighted healthy sleep score.

|  | **Healthy Sleep Score** | | | |  |  |
| --- | --- | --- | --- | --- | --- | --- |
|  | 0~1 | 1~<2.5 | 2.5~<4 | 4~5 | P trend | HR continuous |
| **Microvascular complications** |  |  |  |  |  |  |
| Model1 | Ref | 0.87(0.77 , 0.97) | 0.79(0.71 , 0.88) | 0.76(0.65 , 0.88) | 0.015 | 0.87(0.77 , 0.97) |
| Model2 | Ref | 0.87(0.78 , 0.98) | 0.81(0.73 , 0.91) | 0.77(0.66 , 0.90) | 0.023 | 0.87(0.78 , 0.98) |
| Model3 | Ref | 0.89(0.79 , 0.99) | 0.82(0.74 , 0.92) | 0.80(0.68 , 0.93) | 0.039 | 0.89(0.79 , 0.99) |
| **Cardiovascular outcomes** |  |  |  |  |  |  |
| Model1 | Ref | 0.83(0.72 , 0.96) | 0.76(0.66 , 0.88) | 0.63(0.54 , 0.74) | <0.001 | 0.87(0.84 , 0.91) |
| Model2 | Ref | 0.84(0.73 , 0.97) | 0.80(0.70 , 0.92) | 0.67(0.57 , 0.78) | <0.001 | 0.89(0.85 , 0.93) |
| Model3 | Ref | 0.85(0.74 , 0.98) | 0.81(0.71 , 0.93) | 0.69(0.59 , 0.80) | <0.001 | 0.90(0.86 , 0.94) |

Model1. Age (continuous), Sex (male/female), Ethnic (Whiter/other).

Model2. model1+Activity_MET (low/median/high), The Townsend Deprivation Index (continuous), Alcohol consumption (Special occasions only or Never for yes/no), Smoke status (yes/no).

Model3. model2+TG(continuous), Hypertension (yes/no), Hba1c (continuous).

We constructed a weighted healthy sleep scoree based on the 4 work factors by using the equation: weighted healthy sleep score= (β1×factor1 +β2 ×factor 2 +…+β5×factor 5) × (5/sum of the β coefficients). This weighted score also ranges from 0 to 5 points but considers magnitudes of the adjusted relative risk for each factor in each sleep pattern as a combination of 5 factors.

Supplementary Table6. Associations between healthy sleep score and incident microvascular complications or cardiovascular outcomes after excluding incident cases occurred in the first 2 years of follow-up.

|  | **Microvascular complications** | | | **Cardiovascular outcomes** | | |
| --- | --- | --- | --- | --- | --- | --- |
| Healthy Sleep Score | 0–1 | Ref | HR continuous | 0–1 | Ref | HR continuous |
|  | 2 | 0.88(0.79 , 0.99) | 0.95(0.92 , 0.98) | 2 | 0.86(0.74 , 0.99) | 0.92( 0.88 , 0.96) |
|  | 3 | 0.85(0.76 , 0.94) |  | 3 | 0.87(0.76 , 1.00) |  |
|  | 4 | 0.82(0.73 , 0.91) |  | 4 | 0.73(0.63 , 0.85) |  |

Multivariable-adjusted Model:Age (continuous), Sex (male/female), Ethnic (Whiter/other), Activity_MET (low/median/high), The Townsend Deprivation Index (continuous), Alcohol consumption (Special occasions only or Never for yes/no), Smoke status (yes/no),TG(continuous), Hypertension (yes/no), Hba1c (continuous).

Supplementary Table7. Multivariable-adjusted HRs (95%CIs) for incident microvascular complications or cardiovascular outcomes among 30,915 UK Biobank T2DM participants by different Sleep pattern.

|  | **Sleep pattern** | | |  |  |
| --- | --- | --- | --- | --- | --- |
|  | Poor | Intermediate | Healthy | P trend | HR continuous |
| **Microvascular complications** |  |  |  |  |  |
| Model1 | Ref | 0.83(0.75 , 0.91) | 0.75(0.67 , 0.84) | <0.001 | 0.88(0.84 , 0.93) |
| Model2 | Ref | 0.85(0.77 , 0.94) | 0.78(0.70 , 0.86) | <0.001 | 0.89(0.85 , 0.94) |
| Model3 | Ref | 0.86(0.78 , 0.95) | 0.80(0.72 , 0.89) | <0.001 | 0.91(0.86 , 0.95) |
| **Cardiovascular outcomes** |  |  |  |  |  |
| Model1 | Ref | 0.80(0.71 , 0.91) | 0.65(0.57 , 0.75) | <0.001 | 0.81(0.76 , 0.86) |
| Model2 | Ref | 0.84(0.74 , 0.95) | 0.69(0.60 , 0.79) | <0.001 | 0.83(0.78 , 0.89) |
| Model3 | Ref | 0.85(0.75 , 0.96) | 0.71(0.62 , 0.81) | <0.001 | 0.84(0.79 , 0.90) |

Model1. Age (continuous), Sex (male/female), Ethnic (Whiter/other).

Model2. model1+Activity_MET (low/median/high), The Townsend Deprivation Index (continuous), Alcohol consumption (Special occasions only or Never for yes/no), Smoke status (yes/no).

Model3. model2+TG(continuous), Hypertension (yes/no), Hba1c (continuous).

Supplementary Table8. Associations between healthy sleep score and incident individual microvascular complication or cardiovascular outcome among 30,915 UK Biobank T2DM participants.

|  | **Healthy Sleep Score** | | | |
| --- | --- | --- | --- | --- |
|  | 0–1 | 2 | 3 | 4–5 |
| **Diabetic neuropathy** | Ref | 0.79 (0.64 , 0.99) | 0.76 (0.62 , 0.94) | 0.61 (0.48 , 0.76) |
| **Diabetic kidney disease** | Ref | 0.84 (0.73 , 0.97) | 0.79 (0.69 , 0.91) | 0.72 (0.62 , 0.84) |
| **Diabetic retinopathy** | Ref | 1.03 (0.86 , 1.22) | 1.01 (0.86 , 1.20) | 1.06 (0.89 , 1.26) |
| **Stroke** | Ref | 0.80(0.65 , 1.00) | 0.86(0.70 , 1.06) | 0.79(0.63 , 0.98) |
| **Heart failure** | Ref | 0.78(0.66 , 0.93) | 0.77(0.65 , 0.91) | 0.64(0.53 , 0.76) |
| **coronary heart disease** | Ref | 0.93(0.84 , 1.04) | 0.88(0.79 , 0.97) | 0.73(0.66 , 0.82) |

Model1. Age (continuous), Sex (male/female), Ethnic (Whiter/other).

Model2. model1+Activity_MET (low/median/high), The Townsend Deprivation Index (continuous), Alcohol consumption (Special occasions only or Never for yes/no), Smoke status (yes/no).

Model3. model2+TG(continuous), Hypertension (yes/no), Hba1c (continuous).

CI, confidence interval, CVD, cardiovascular disease, HR, hazard ratio, PAF, population-attributable fraction, T2DM, type 2 diabetes mellitus, HbA1c: glycated hemoglobin, TG: total triglyceride, MET:metabolic equivalent task.

Supplementary Table9. HRs of complications by individual healthy sleep behaviors among 30,915 UK Biobank T2DM participants .

|  | **Cardiovascular outcomes** | | **Microvascular complications** | |
| --- | --- | --- | --- | --- |
|  | HR (95% CI) | p-value | HR (95% CI) | p-value |
| **No self-report snoring** | 0.96 (0.89 , 1.03) | 0.292 | 0.99(0.94 , 1.05) | 0.851 |
| **No frequent insomnia** | 0.87(0.79 , 0.95) | 0.003 | 0.94(0.87 , 1.01) | 0.071 |
| **No frequent daytime sleepiness** | 0.74(0.64 , 0.85) | <0.001 | 0.85(0.75 , 0.96) | 0.009 |
| **Early chronotype** | 0.96(0.89 , 1.04) | 0.295 | 0.95(0.90 , 1.01) | 0.108 |
| **Sleep 7-8 h/d** | 0.84(0.78 , 0.90) | <0.001 | 0.90 (0.85 , 0.95) | <0.001 |

Multivariable-adjusted Model:Age (continuous), Sex (male/female), Ethnic (Whiter/other), Activity_MET (low/median/high), The Townsend Deprivation Index (continuous), Alcohol consumption (Special occasions only or Never for yes/no), Smoke status (yes/no),TG(continuous), Hypertension (yes/no), Hba1c (continuous).
